# Recombinant human thrombopoietin for chronic liver disease-associated thrombocytopenia with or without concomitant infections: a real-world retrospective study

**DOI:** 10.3389/fphar.2026.1732969

**Published:** 2026-03-23

**Authors:** Xun Wei, Li Ma, Wen Bo Liu, Ya Mei Kuang, Xin Meng Qin, Jun Feng Wei, Yi Kang

**Affiliations:** Department of Infectious Diseases, People’s Hospital of Zhengzhou University, Zhengzhou, China

**Keywords:** chronic liver disease, concomitant infection, platelet count, recombinant human thrombopoietin, thrombocytopenia

## Abstract

**Objectives:**

Recombinant human thrombopoietin (rhTPO) has been shown to improve platelet (PLT) counts in chronic liver disease (CLD)-associated thrombocytopenia (TP). However, whether concomitant infections influence its efficacy remains unclear.

**Methods:**

We conducted a retrospective study of 259 patients with CLD-associated TP who received rhTPO at Henan Provincial People’s Hospital from January 2021 to October 2023. Patients were divided into concomitant infection (n = 178) and non-concomitant infection (n = 81) groups. Primary endpoints were the response rate and time to response. Secondary endpoints included the platelet transfusion requirements, PLT recovery differences and factors influencing rhTPO efficacy. Propensity score matching (PSM) was applied to adjust for baseline confounders.

**Results:**

After 1:1 PSM (n = 132), the overall response rate was 57.6% and the median time to response was 10 days, with no serious adverse events observed. Response rates and response time were comparable between concomitant infection and non-concomitant infection groups (59.1% vs. 56.1%, *P* = 0.725; 10 days vs. 11 days, *P* = 0.442). rhTPO significantly increased PLT counts from Day 3 in patients without concomitant infection and from Day 5 in those with concomitant infection. Both groups maintained elevated levels at 14 days post-discontinuation and remained above baseline until 28 days after discontinuation. No significant differences between the two groups were observed at any time point. Patients with concomitant infection had a significant higher platelet transfusion rate than those without infections (22.7% vs. 7.6%, *P* = 0.015). Sensitivity analysis excluding these transfused patients showed consistent efficacy of rhTPO regardless of infection status. Multivariable analyses identified Child–Pugh C and cirrhosis as independent factors associated with response and time to response.

**Conclusion:**

rhTPO was associated with improved PLT counts in CLD-associated TP without serious adverse events. This efficacy appeared comparable regardless of concomitant infection status. Liver function reserve may be the major determinant of efficacy. Prospective multicenter studies are needed to confirm these findings.

## 1 Introduction

Chronic liver disease (CLD) is defined as liver damage persisting for more than 6 months. As reported by the Global Burden of Disease estimates, CLD is responsible for approximately two million deaths worldwide annually. Nearly half of these deaths occur in the Asia-Pacific region, which bears a substantial proportion of the global burden of CLD (Devarbhavi et al., 2023; Mak et al., 2024). Cirrhosis represents the end stage of CLD, characterized by disruption of liver architecture, formation of widespread nodules, vascular remodeling and angiogenesis (Li, 2021; Somnay et al., 2024). These alterations can trigger multiple downstream complications, including portal hypertension, hemostatic imbalance, and hematological abnormalities.

Thrombocytopenia (TP) is one of the most common hematological complications in CLD. Its prevalence ranges from 6% in non-cirrhotic CLD patients to 78% in those with advanced cirrhosis (Qian et al., 2021; Saab and Brown, 2019; Gangireddy et al., 2014; Maan et al., 2015; Scharf, 2021). Severe TP increases the bleeding risk, complicates clinical management and potentially results in treatment delays or cancellation. Currently, the pathogenesis of CLD-associated TP is considered to be multifactorial, encompassing hypersplenism induced by portal hypertension, impaired production of thrombopoietin (TPO), myelosuppression and accelerated platelet (PLT) destruction (Mitchell et al., 2016; Miller et al., 2019; Peck-Rad and osavljevic, 2017). Despite low PLT counts, patients with advanced CLD often exhibit a relatively procoagulant state (Roberts, 2021). This paradoxical phenomenon has been attributed to elevated levels of von Willebrand factor (vWF) and reduced ADAMTS13. Consequently, while the PLT count declines, the PLT functional activity is enhanced, leading to a rebalanced hemostasis profile (Roberts, 2021; Hugenholtz et al., 2013). This fragile balance can be disrupted by external factors such as infection. A previous study reported that 47% of CLD patients suffered from concomitant infections, which may further reduce PLT levels and worsen clinical outcomes (Foreman et al., 2003).

PLT transfusion is the main clinical intervention for CLD patients with TP (Qian et al., 2021). However, it has several limitations, including the risk of infusion reactions and infections, the development of anti-platelet antibodies, short storage time, and potential lack of platelet sources (Solves Alcaina, 2020; Blumberg et al., 2010). In addition, patients who received repeated transfusions may become refractory to subsequent PLT support (Stanworth et al., 2015). Therefore, there remains an urgent clinical need for alternative therapeutics that can rapidly and sustainably elevate PLT levels. Recombinant human thrombopoietin (rhTPO) offers a promising therapeutic strategy for patients with CLD-associated TP.

rhTPO is structurally similar to endogenous TPO, which specifically promotes the proliferation and maturation of megakaryocytes to increase PLT levels (Kuter, 2013; Wörmann, 2013). In China, it has been approved for chemotherapy-induced TP (CIT) (Consensus Committee of Chemotherapy Induced Thrombocytopenia and Chinese Society of Clinical Oncology, 2018) and immune thrombocytopenia (ITP) (Liu et al., 2018). Recently, rhTPO was approved for CLD-associated TP in patients undergoing invasive procedures based on a Phase 3 trial in China (CTR20230919).

Given the high prevalence of concomitant infections in CLD patients, which makes clinical management more complicated. Inflammation may further suppress bone marrow function, raising uncertainty regarding the efficacy of rhTPO. Patients with concomitant infections were usually excluded from clinical trials of thrombopoietin receptor agonists, such as the phase III trial of avatrombopag (Poordad et al., 2020), leaving an evidence gap in clinical practice. Therefore, this retrospective real-world study evaluated the efficacy of rhTPO in CLD patients with TP, focusing on comparing outcomes between those with and without concomitant infections. We aimed to provide preliminary clinical insights that may inform treatment strategy for this subgroup.

## 2 Materials and methods

### 2.1 Subjects

**FIGURE 1:**
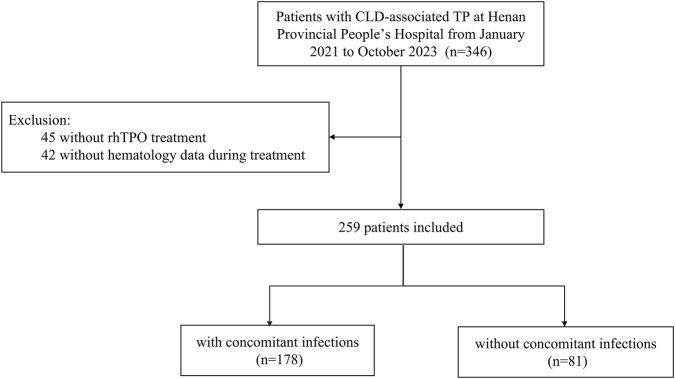
Flow diagram of the population enrolled.

This retrospective study included patients with CLD-associated TP admitted to the Department of Infectious Diseases at Henan Provincial People’s Hospital from January 2021 to October 2023. A total of 259 patients were included and divided into two groups: CLD-associated TP without concomitant infection (n = 81) and CLD-associated TP with concomitant infection (n = 178) (Figure 1). Concomitant infection was diagnosed based on microbiological evidence, clinical and imaging findings, and elevated inflammatory markers, while sepsis was defined in accordance with the Sepsis-3 criteria (Singer et al., 2016).

The inclusion criteria were: (1) diagnosis of chronic hepatitis, liver cirrhosis, or liver cancer for ≥6 months; (2) age >18 years; (3) PLT <75 × 10^9^/L; (4) hospitalization >7 days, with available follow-up data at least 1month after treatment discontinuation. The exclusion criteria were: (1) patients who used platelet inhibitors in the past 6 months or suffered from thrombotic disorders; (2) patients with malignancies undergoing oncological treatments; (3) patients with haematological or immune system diseases (excluding autoimmune hepatitis), and/or with pseudo- or congenital TP; (4) patients who underwent splenectomy or liver transplantation within 6 months before or after treatment; (5) patients allergic to rhTPO; (6) women of childbearing potential who were pregnant, lactating, or not using effective contraception; and (7) patients concurrently using other drugs, such as steroids, that affect the PLT.

This study was approved by the Ethics Committee of Henan Provincial People’s Hospital [Ethical Review No. (2024)86].

### 2.2 Treatment

All patients received symptomatic and supportive care. rhTPO was administered subcutaneously at 300 U/kg once a day, with a maximum treatment duration of 14 days. Treatment was discontinued when PLT increased by ≥ 50 × 10^9^/L from baseline, or PLT ≥100 × 10^9^/L on two consecutive measurements within 14 days. For patients receiving combination therapy with rhTPO and avatrombopag, avatrombopag was administered orally at 40–60 mg/day for 5 days. Platelet transfusion was used as a rescue therapy in patients with severe thrombocytopenia (PLT counts< 30 × 10^9^/L), active bleeding, or preparation for high risk invasive procedures.

### 2.3 Data collection

Baseline and follow-up data were retrospectively extracted from electronic medical records, laboratory and imaging reports. We collected baseline characteristics (sex, age, body mass index (BMI), diagnosis, disease duration), laboratory parameters (PLT, albumin, alanine transaminase (ALT), aspartate transaminase (AST), alkaline phosphatase (ALP), total bilirubin (TBIL), serum albumin, urea, creatinine levels, prothrombin time, prothrombin time activity), treatment types and platelet transfusion information.

PLT counts were collected on day 1, 3, 5, 7, and 14 after treatment initiation, and on day 14 and 28 after rhTPO discontinuation (±1 day window). Safety events were monitored from treatment initiation to 1 month after rhTPO discontinuation.

### 2.4 Study endpoints

The primary efficacy endpoints were defined as treatment response rate and time to response. A response was achieved when post-treatment platelet counts reached ≥50 × 10^9^/L (with a ≥10 × 10^9^/L absolute increase) in patients with baseline <50 × 10^9^/L, or ≥100 × 10^9^/L in those with baseline 50 - 75 × 10^9^/L. Time to response was defined as the days from treatment initiation to first reach response.

The secondary endpoints included the platelet transfusion, PLT recovery differences between concomitant and non-concomitant infections and exploratory analysis for independent factors influencing efficacy.

### 2.5 Statistical analysis

All data were analyzed using R (version 4.3.3). Continuous variables are presented as mean ± standard deviation (SD) or median (interquartile range (IQR)). For normally distributed data, comparisons between the two groups were using t-test, while the Wilcoxon rank-sum test was used for non-normally distributed data. Categorical variables are presented as n (%), with chi-squared or Fisher’s exact test used for comparisons.

Propensity score matching (PSM) was performed using the MatchIt package (version 4.5.5) with 1:1 nearest neighbor matching and a caliper of 0.2. Baseline clinical variables, including age, sex, cirrhosis, Child-Pugh score and baseline PLT count, were included as covariates. Balance was assessed using standardized mean differences (SMDs). PLT dynamic changes were visualized using the ggplot2 package (version 3.5.2). Univariable and multivariable logistic regression analyses were conducted with the base R function glm. Multicollinearity was assessed using variance inflation factors (VIF), with VIF <5 considered acceptable. Univariable and multivariable Cox proportional hazards regression were performed using the survival package (version 3.5.8). The proportional hazards (PH) assumption was tested using scaled Schoenfeld residuals. Forest plots were generated using the forestploter package (version 1.1.2). All statistical tests were two-sided. Statistical significance was determined at *P* < 0.05.

## 3 Results

### 3.1 Demographic and clinical characteristics

A total of 259 patients with CLD-associated TP were enrolled in this study, comprising 178 (68.7%) patients with concomitant infection and 81 (31.3%) without concomitant infection. Among the concomitant infected group, 94.4% (168/178) had bacterial infections and 32% (57/178) developed sepsis. Importantly, no bleeding or thrombosis events were observed during the study period.

Baseline characteristics are shown in Table 1. Before PSM, significant imbalances were observed between the two groups. The concomitant infected group was older (55.5 vs. 52.0 years, *P* = 0.03) and had more male patients (74.2% vs. 51.9%, *P* < 0.001). This group also exhibited more severe liver disease status, characterized by significantly higher rates of liver failure (46.6% vs. 22.2%, *P* < 0.001), higher Child-Pugh grades (*P* < 0.001), lower albumin (*P* = 0.013), and elevated creatinine (*P* = 0.019). Treatment regimens also differed. Combination therapy (rhTPO plus avatrombopag) was more common in the non-concomitant infected group (34.6% vs. 20.8%, *P* = 0.018). No significant difference was observed between the groups in baseline PLT (*P* = 0.132).

**TABLE 1:** Comparisons of baseline characteristics of CLD-associated TP patients with or without concomitant infections before PSM.

Characteristic	Overall (n = 259)	Non-concomitant infection (n = 81)	Concomitant infection (n = 178)	*P* value
Demographics				
Age (years), median (IQR)	54.00 (48.00–62.00)	52.00 (47.00–58.00)	55.50 (48.00–64.00)	0.030
Female sex, n (%)	85 (32.8)	39 (48.1)	46 (25.8)	<0.001
BMI (kg/m^2^), median (IQR)	23.23 (20.82–26.16)	23.57 (21.48–26.18)	22.97 (20.55–26.16)	0.228
Liver-related features				
Cirrhosis, n (%)	236 (91.1)	79 (97.5)	157 (88.2)	0.014
Liver tumor, n (%)	41 (15.8)	9 (11.1)	32 (18.0)	0.160
Liver failure, n (%)	101 (39.0)	18 (22.2)	83 (46.6)	<0.001
Child-Pugh grades, n (%)	​	​	​	<0.001
Grade a	61 (23.6)	35 (43.2)	26 (14.6)	​
Grade B	129 (49.8)	38 (46.9)	91 (51.1)	​
Grade C	69 (26.6)	8 (9.9)	61 (34.3)	​
Albumin (g/L), median (IQR)	30.20 (27.25–33.00)	31.02 (28.80–34.60)	29.75 (26.70–32.70)	0.013
ALT (U/L), median (IQR)	31.00 (19.10–54.05)	28.60 (19.10–46.30)	31.60 (19.13–56.28)	0.334
AST (U/L), median (IQR)	43.30 (28.50–70.65)	39.00 (28.60–60.60)	44.40 (28.55–80.45)	0.216
ALP (U/L), median (IQR)	99.50 (74.40–148.75)	98.90 (73.30–152.10)	99.55 (75.45–147.15)	0.694
Infection characteristics				
Infection type, n (%)	​	​	​	-
Bacterial-involved^a^	-	​	168 (94.4)	​
Others^b^	-	​	10 (5.6)	​
Sepsis, n (%)	--	-	57 (32.0)	-
Laboratory parameters				
PLT count (10^9^/L), median (IQR)	32.00 (24.00–45.00)	34.00 (25.00–48.00)	32.00 (23.00–44.00)	0.132
Creatinine (μmol/L), median (IQR)	57.40 (47.00–86.00)	55.00 (46.00–64.75)	60.50 (47.25–101.75)	0.019
Treatment regimen				
Treatment, n (%)	​	​	​	0.018
rhTPO plus avatrombopag	65 (25.1)	28 (34.6)	37 (20.8)	​
rhTPO	194 (74.9)	53 (65.4)	141 (79.2)	​
Treatment duration (days), median (IQR)	8.0 (5.0–11.0)	9.0 (6.0–11.0)	8.0 (5.0–11.0)	0.064

^a^ Bacterial-involved includes bacterial, bacterial + viral, and bacterial + fungal infections.

^b^ Others include viral, fungal, and unknown pathogens.

Abbreviations: BMI, body mass index; IQR, interquartile range; ALT, alanine transaminase; AST, aspartate transaminase; ALP, alkaline phosphatase.

After 1:1 PSM, 66 pairs were matched. Liver function and treatment were balanced between the two groups (Supplementary Table S1; Supplementary Figure S1). Therefore, these matched 132 patients were used in subsequent analyses.

### 3.2 Primary efficacy endpoints

**FIGURE 2:**
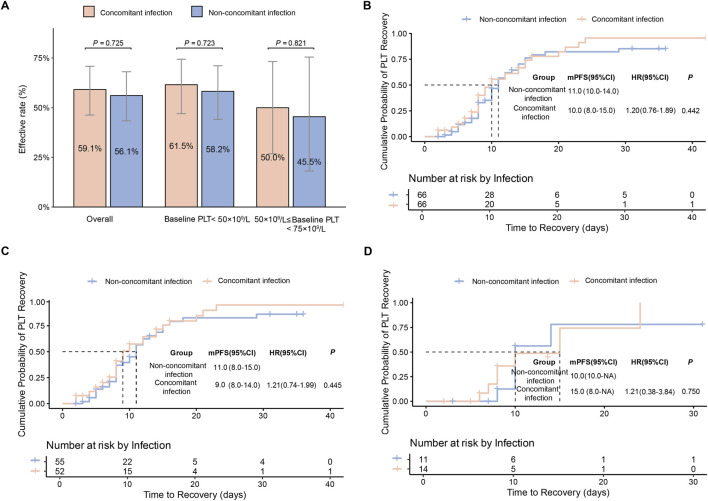
Primary efficacy endpoints in the PSM cohort **(A)** Effective rate in the overall population and in baseline PLT subgroups, stratified by concomitant infection. Data are presented as percentages with 95% confidence intervals (CI). **(B)** Comparison of PLT recovery time in the overall population. **(C)** Comparison of PLT recovery time in patients with baseline PLT<50 × 10^9^/L. **(D)** Comparison of PLT recovery time in patients with 50 × 10^9^/L≤ baseline PLT<75 × 10^9^/L.

In the PSM cohort, the overall response rate was 57.6% (76/132), with 59.1% (39/66) in the concomitant infected group and 56.1% (37/66) in the non-concomitant infected group (Figure 2A). Median time to response was 10 days and 11 days, respectively (Figure 2B).

Subgroup analyses by baseline PLT (<50 × 10^9^/L and 50 - 75 × 10^9^/L) also showed no significant difference between the two groups. Among patients with PLT <50 × 10^9^/L, response rates were 61.5% (32/52) in the concomitant infected group and 58.2% (32/55) in the non-concomitant infected group (Figure 2A), with median time to response of 9 days and 11 days (Figure 2C), respectively. Among patients with baseline PLT between 50 × 10^9^/L and 75 × 10^9^/L, response rates were 50% (7/14) in the concomitant infection group and 45.5% (5/11) in the non-concomitant infection group (Figure 2A), with median time to response of 15 and 10 days, respectively (Figure 2D). Analyses in the full cohort yielded similar results (Supplementary Table S2).

### 3.3 PLT dynamic changes during the study period

**FIGURE 3:**
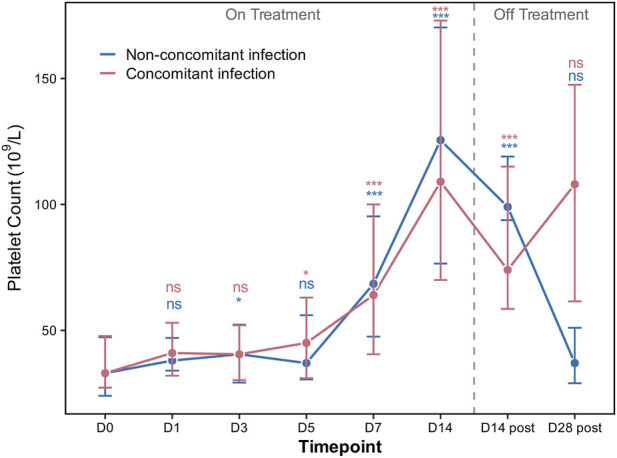
Dynamic changes in PLT counts during and after rhTPO treatment in the PSM cohort.

Dynamic PLT changes were assessed during treatment (Day 1, 3, 5, 7, 14) and at 14 and 28 days after discontinuation in the PSM cohort (Figure 3). In the concomitant infection group, PLT significantly increased from Day 5 and remained elevated through Day14 during treatment. In the non-concomitant infection group, PLT significantly increased from Day 3. Although no significant difference was observed at Day 5, PLT continued to rise throughout the treatment period.

After treatment cessation, both groups exhibited decrease in PLT at 14 days post-discontinuation but remained significantly higher than baseline until 28 days post-discontinuation. There were no significant differences between the two groups at any time point. Overall, these observations suggest that rhTPO induced a rapid and sustained increase in PLT counts regardless of concomitant infection status.

### 3.4 PLT transfusion

In the PSM cohort, 22.7% (15/66) patients in the concomitant infection group received platelet transfusions, compared with 7.6% (5/66) in the non-concomitant infection group (*P* = 0.015). Among transfused patients, the median number of transfusions (2.0 vs. 2.0, *P* = 0.26) and transfusion volumes (2.0 vs. 2.0 units, *P* = 0.26) were similar between the two groups (Table 2). In the full cohort, the platelet transfusion rate remained significantly higher in the concomitant infection group (32.6% vs. 8.6%, *P* < 0.001).

**TABLE 2:** Platelet transfusion in PSM cohort.

Characteristics	Total (n = 132)	Concomitant infection (n = 66)	Non-concomitant infection (n = 66)	*P* value
Platelet transfusion (n, %)				0.015
Yes	20 (15.2)	15 (22.7)	5 (7.6)	​
No	112 (84.8)	51 (77.3)	61 (92.4)	​
Among transfused patients				
Number of platelet transfusions, median (IQR)	2.0 (1.0–3.0)	2.0 (1.0–3.0)	2.0 (1.0–2.0)	0.256
Platelet transfusion volume (U), median (IQR)	2.0 (1.0–3.0)	2.0 (1.0–3.0)	2.0 (1.0–2.0)	0.256

### 3.5 Sensitivity analysis excluding transfused patients in the PSM cohort

**FIGURE 4:**
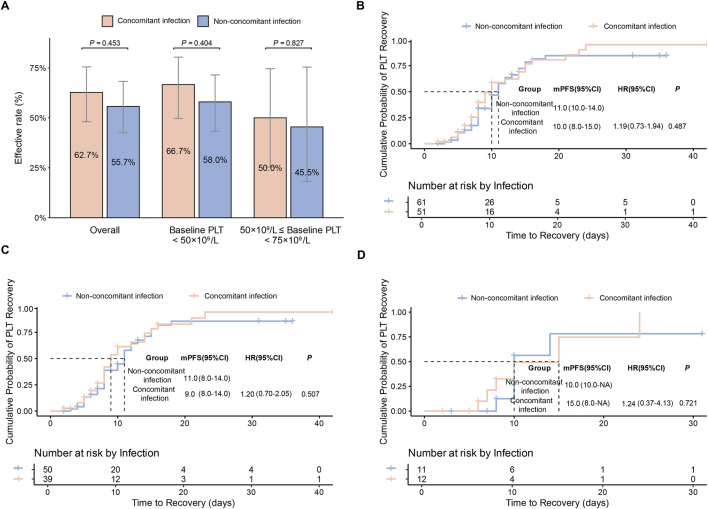
Sensitivity analysis of primary efficacy endpoints in the PSM cohort excluding patients with platelet transfusions. **(A)** Effective rate in the overall population and in baseline PLT subgroups, stratified by concomitant infection. Data are presented as percentages with 95% confidence intervals (CI). **(B)** Comparison of PLT recovery time in the overall population. **(C)** Comparison of PLT recovery time in patients with baseline PLT<50 × 10^9^/L. **(D)** Comparison of PLT recovery time in patients with 50 × 10^9^/L≤ baseline PLT<75 × 10^9^/L.

To exclude the confounding effect of platelet transfusion, we excluded patients who received platelet transfusions in the matched cohort. The remaining patients (n = 51 vs. 61) were further included in the primary efficacy analysis, which also showed no significant differences between the two groups in overall response rate (62.7% [32/51] vs. 55.7% [34/61], *P* = 0.453) (Figure 4A) or time to response (median 10 days vs. 11 days, log-rank *P* = 0.487) (Figure 4B). Subgroup analyses (both baseline PLT<50 × 10^9^/L and 50 × 10^9^/L - 75 × 10^9^/L further showed no significantly difference between groups (*P* > 0.05) (Figures 4C,D). Taken together, these exploratory analyses suggest that no detectable efficacy differences between the concomitant infection and non-concomitant infection groups.

### 3.6 Univariable and multivariable analyses for rhTPO efficacy

To explore the factors influencing the response rate and time to response of rhTPO, we conducted univariate logistic regression and univariate Cox regression analyses in the overall cohort. Baseline PLT count was significantly associated with rhTPO efficacy (Table 3).

**TABLE 3:** Univariable logistic and cox regression analyses for treatment response and time to response in the entire cohort (N = 259).

Factors	n	Univariable logistic regression (response Rate)		Univariable cox regression (time to response)	
		Or (95% CI)	*P* value	HR (95% CI)	*P* value
Age					
≥50 vs. <50	176 vs. 83	1.22 (0.72–2.06)	0.464	1.27 (0.90–1.80)	**0.175**
Sex					
Male vs. Female	174 vs. 85	0.99 (0.58–1.68)	0.975	0.9 (0.64–1.26)	**0.527**
BMI					
≥24 vs. <24	220 vs. 22	1.23 (0.50–2.96)	0.650	1.41 (0.78–2.55)	**0.254**
Cirrhosis					
Yes vs. No	236 vs. 23	0.47 (0.17–1.18)	0.127	0.51 (0.31–0.85)	**0.009**
Liver tumor					
Yes vs. No	41 vs. 218	1.43 (0.72–2.96)	0.311	1.04 (0.69–1.58)	**0.854**
Liver failure					
Yes vs. No	101 vs. 158	0.80 (0.48–1.33)	0.397	1.23 (0.88–1.71)	**0.222**
Child-Pugh grades					
Grade B vs. Grade A	129 vs. 61	0.96 (0.51–1.78)	0.889	0.98 (0.67–1.45)	**0.929**
Grade C vs. Grade A	69 vs. 61	0.62 (0.31–1.25)	0.186	0.76 (0.48–1.21)	**0.250**
Concomitant infection					
Yes vs. No	178 vs. 81	1.12 (0.66–1.90)	0.676	1.03 (0.72–1.45)	**0.887**
Sepsis					
Yes vs. No	57 vs. 200	1.40 (0.76–2.61)	0.286	0.98 (0.68–1.42)	**0.925**
Baseline PLT count					
≥50 vs. <50	45 vs. 214	0.5 (0.26–0.95)	0.035	0.67 (0.42–1.08)	**0.101**
Treatment					
rhTPO vs. rhTPO plus avatrombopag	194 vs. 65	0.93 (0.52–1.64)	0.804	1.48 (1.03–2.13)	**0.036**

Abbreviations: OR, odd ratio; HR, hazard ratio; CI, confidence interval. Bold values indicate P < 0.05.

Given that the definition of response differed by baseline PLT, we stratified data according to baseline PLT status and performed multivariable logistic and cox regression analyses. Variables with *P* < 0.2 and clinically important factors, were included in multivariate models.

**FIGURE 5:**
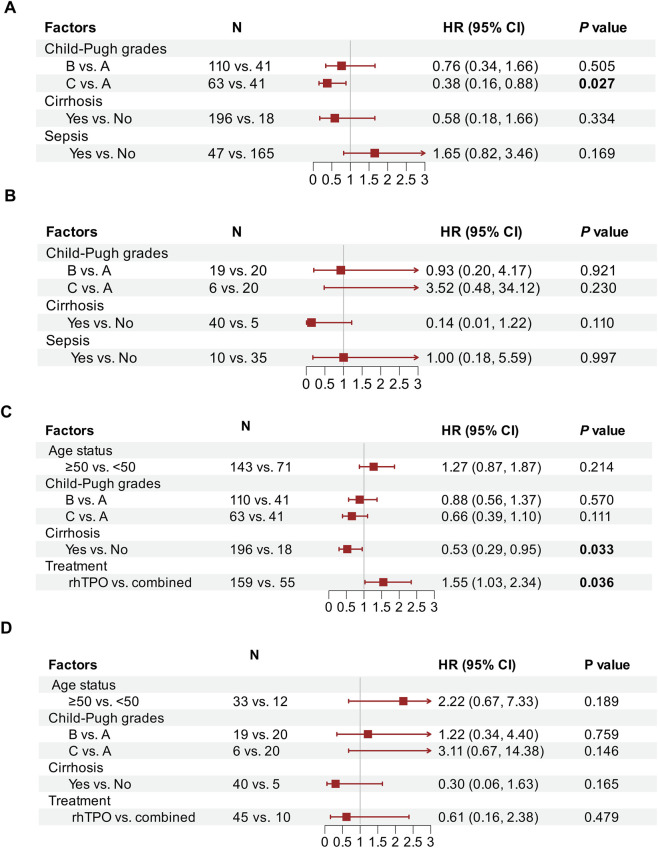
Multivariable logistic and cox regression analyses for treatment response and time to response in the entire cohort: **(A)** Multivariable logistic for subgroup with a baseline platelet count <50 × 10^9^/L; **(B)** Multivariable logistic for subgroup with 50 × 10^9^/L ≤ Baseline PLT <75 × 10^9^/L; **(C)** Multivariable cox for subgroup with a baseline platelet count <50 × 10^9^/L; **(D)** Multivariable cox for subgroup with 50 × 10^9^/L ≤ Baseline PLT <75 × 10^9^/L.

Regarding the response rate, in patients with baseline PLT <50 × 10^9^/L, Child-Pugh C was identified as an independent risk factor (OR = 0.38, 95%CI: 0.16–0.88) (Figure 5A). In patients with baseline PLT ≥50 × 10^9^/L, cirrhosis showed a tend towards lower response rate, although the statistical significance was not reached due to the relatively small sample size (Figure 5B).

As for time to response, in patients with a baseline PLT <50 × 109/L, cirrhosis and treatment regimen were identified as two independent factors. Cirrhosis prolonged the time to response, while rhTPO monotherapy significantly shortened the time to response (Figure 5C). In patients with baseline PLT ≥50 × 10^9^/L, cirrhosis also showed a trend toward delayed response (Figure 5D).

## 4 Discussion

In this retrospective study of 259 patients with CLD-associated TP, rhTPO treatment was observed to be effective and well tolerated. PLT levels significantly increased from Day 3 in patients without concomitant infection and from Day 5 in those with concomitant infection. In both groups, PLT remained above baseline at 14 days post-discontinuation until 28 days post-discontinuation, indicating a rapid onset and sustained effect of rhTPO. No serious AEs, such as bleeding or thrombosis, were documented. Importantly, concomitant infection—including sepsis—did not influence the efficacy of rhTPO, with response rates of approximately 55%–60% in both groups.

Previous studies have confirmed that rhTPO is effective and safe for treating patients with CLD-related TP. In a multicenter observational study of HBV-associated TP, rhTPO significantly increases PLT counts and reduced bleeding risk (Zhai et al., 2018). More recently, a prospective trial reported a 60.7% response rate of rhTPO in acute-on-chronic liver failure, which was comparable with our study (Liu et al., 2025). In sepsis-associated TP, rhTPO administration in severe sepsis significantly improved PLT counts and reduced platelet transfusion requirements (Wu et al., 2014). It also mitigated endothelial injury and inflammatory markers (Xie et al., 2024) and improved prognosis in sepsis (Zhou et al., 2020). Our exploratory results are consistent with these reports and extend them to CLD-associated TP with concomitant infection.

Mechanistically, TPO is mainly synthesized and secreted by the liver and released into the blood. TPO binds to the c-MPL receptor on platelets, haematopoietic stem, progenitor cells, and megakaryocytes. This interaction activates intracellular signaling pathways to promote megakaryocytes maturation and platelet production (Saur et al., 2010; Sattler et al., 1995; Rouyez et al., 1997). While in CLD patients, liver parenchymal destruction reduces TPO mRNA expression and synthesis, lowering PLT levels and causing TP in CLD patients, aside from hypersplenism (Ishikawa et al., 1998; Goulis et al., 1999). Supplementation with exogenous rhTPO can compensate for its deficiency and restore platelet recovery.

We further investigated clinical factors influencing response and time to response. According to previous clinical studies, moderate (20 - 50 × 10^9^/L) or severe (<20 × 10^9^/L) thrombocytopenia predicts poor outcomes in advanced CLD (Bleibel et al., 2013). In our cohort, 82.6% (214/259) of patients had baseline PLT <50 × 10^9^/L. Multivariable logistic regression identified Child-Pugh score as the independent factor associated with response, with efficacy declined progressively from class A to C. Multivariable Cox model showed that cirrhosis independently prolonged time to response. Even among patients with baseline PLT ≥50 × 10^9^/L, cirrhosis showed a trend toward reduced response rate. These findings suggest compared with concomitant infection, hepatic functional reserve may be more closely affect rhTPO efficacy in CLD-associated TP, validating the rationale for PSM. Of note, rhTPO monotherapy achieved target PLT counts faster than combination therapy. This unexpected finding may reflect treatment selection bias.

Due to the retrospective design, our study could not capture all treatment-emergent AEs, especially for mild AEs. Nevertheless, the safety profile of rhTPO has been established in other populations, including CIT and ITP (Chen et al., 2025; Xu et al., 2018). The most commonly reported AEs are generally mild and transient, such as fever, fatigue, and mild injection-site reactions. In a prior prospective study of advanced CLD, rhTPO was not associated with an increased incidence of thrombotic events and bleeding rates were numerically lower (Liu et al., 2025). In our cohort, based on available medical records, no major safety signals, such as severe bleeding or thrombosis, were documented during the study period. This observation is consistent with the established safety data and supports the favorable risk-benefit profile of rhTPO in the management of CLD-associated TP.

This study has several limitations. First, as a single-center retrospective study, potential selection bias and confounding factors cannot be fully eliminated. Although PSM minimized baseline imbalances, the reduced sample size after matching may limit the statistical power. Therefore, our findings should be interpreted as exploratory. In addition, AEs may have been underestimated due to the retrospective design. Second, the use of avatrombopag was determined by physician discretion, which may introduce treatment heterogeneity and selection bias. Third, the impact of infection severity, particularly sepsis, could not be fully evaluated due to the relatively small number of sepsis patients. Finally, as rhTPO is currently used primarily in China, the generalizability of our results may be limited. Larger multicenter prospective and international studies are needed to validate these exploratory findings.

## 5 Conclusion

In conclusion, rhTPO was associated with improved PLT counts in CLD-associated TP patients, with no serious AEs were observed. Treatment response appeared comparable regardless of concomitant infection status. Liver function reserve may be the major determinant of efficacy. Prospective multicenter studies are needed to confirm these findings.

## Data Availability

The original contributions presented in the study are included in the article/Supplementary Material, further inquiries can be directed to the corresponding author.
