# Supplementary material for: Recombinant human thrombopoietin for chronic liver disease-associated thrombocytopenia with or without concomitant infections: a real-world retrospective study
Source: Front Pharmacol. 2026 Mar 23;17:1732969. doi: 10.3389/fphar.2026.1732969 (PMC13050849; doi:10.3389/fphar.2026.1732969)
Supplement: Supplementary file 1 [file Table1.docx]

**Table S1 Comparison of the baseline characteristics of CLD-associated TP patients with or without** **concomitant** **infections** **after PSM**

| **Characteristic** | **Overall**  **(n=132)** | **Non-concomitant infection (n=66)** | **Concomitant infection (n=66)** | ***P* value** |
| --- | --- | --- | --- | --- |
| **Demographics** |  |  |  |  |
| Age (years), median(IQR) | 52.00 (46.75 – 59.00) | 50.50 (46.25 – 56.00) | 54.50 (48.25 – 60.00) | 0.190 |
| Female Sex, n (%) | 49 (37.1) | 26 (39.4) | 23 (34.8) | 0.589 |
| BMI (kg/m²), median(IQR) | 23.23 (20.95 – 26.08) | 23.83 (21.48 – 26.35) | 22.84 (20.26 – 25.52) | 0.158 |
| **Liver-related Features** |  |  |  |  |
| Cirrhosis, n (%) | 127 (96.2) | 64 (97.0) | 63 (95.5) | >0.99 |
| Liver tumor, n (%) | 18 (13.6) | 6 (9.1) | 12 (18.2) | 0.128 |
| Liver failure, n (%) | 41 (31.1) | 18 (27.3) | 23 (34.8) | 0.347 |
| Child-Pugh grades, n (%) |  |  |  | 0.925 |
| Grade A | 38 (28.8) | 20 (30.3) | 18 (27.3) |  |
| Grade B | 78 (59.1) | 38 (57.6) | 40 (60.6) |  |
| Grade C | 16 (12.1) | 8 (12.1) | 8 (12.1) |  |
| Albumin (g/L), median (IQR) | 30.80 (28.35 – 33.20) | 30.95 (28.72 – 33.15) | 30.50 (27.90 – 33.30) | 0.962 |
| ALT (U/L), median (IQR) | 28.05 (18.38 – 46.68) | 28.40 (19.10 – 46.53) | 26.95 (17.48 – 46.18) | 0.423 |
| AST (U/L), median (IQR) | 41.95 (27.55 – 65.30) | 40.15 (28.63 – 63.38) | 42.75 (25.33 – 68.08) | 0.785 |
| ALP (U/L), median (IQR) | 99.20 (75.18 – 146.70) | 101.40 (75.20 – 163.70) | 98.80 (72.23 – 143.78) | 0.598 |
| **Infection Characteristics** |  |  |  |  |
| Infection type, n (%) |  |  |  | - |
| Bacterial-involved^#^ | - | - | 63 (95.5) |  |
| Others^*^ | - | - | 3 (4.5) |  |
| Sepsis, n (%) | 16 (12.3) | 0 (0.0) | 16 (24.2) | - |
| **Laboratory Parameters** |  |  |  |  |
| PLT count (10^9^/L), median (IQR) | 33.00 (25.75 – 48.00) | 33.00 (24.00 – 47.25) | 33.00 (27.25 – 47.75) | 0.575 |
| Creatinine (μmol/L), median (IQR) | 57.00 (46.25 – 75.00) | 55.50 (46.00 – 65.25) | 57.00 (47.00 – 84.75) | 0.276 |
| **Treatment Regimen** |  |  |  |  |
| Treatment, n (%) |  |  |  | 0.104 |
| rhTPO plus avatrombopag | 32 (24.2) | 20 (30.3) | 12 (18.2) |  |
| rhTPO | 100 (75.8) | 46 (69.7) | 54 (81.8) |  |
| Treatment duration (days), median (IQR) | 8 (6–11) | 8 (6–11) | 8 (6–11) | 0.433 |

^#^ Bacterial-involved includes bacterial, bacterial + viral, and bacterial + fungal infections.

* Others include viral, fungal, and unknown pathogens.

**Abbreviations**: BMI, body mass index; IQR, interquartile range; ALT, Alanine transaminase; AST, Aspartate transaminase; ALP: Alkaline phosphatase.
