# Supplementary figures and images for: Recombinant human thrombopoietin for chronic liver disease-associated thrombocytopenia with or without concomitant infections: a real-world retrospective study
Source: Front Pharmacol. 2026 Mar 23;17:1732969. doi: 10.3389/fphar.2026.1732969 (PMC13050849; doi:10.3389/fphar.2026.1732969)

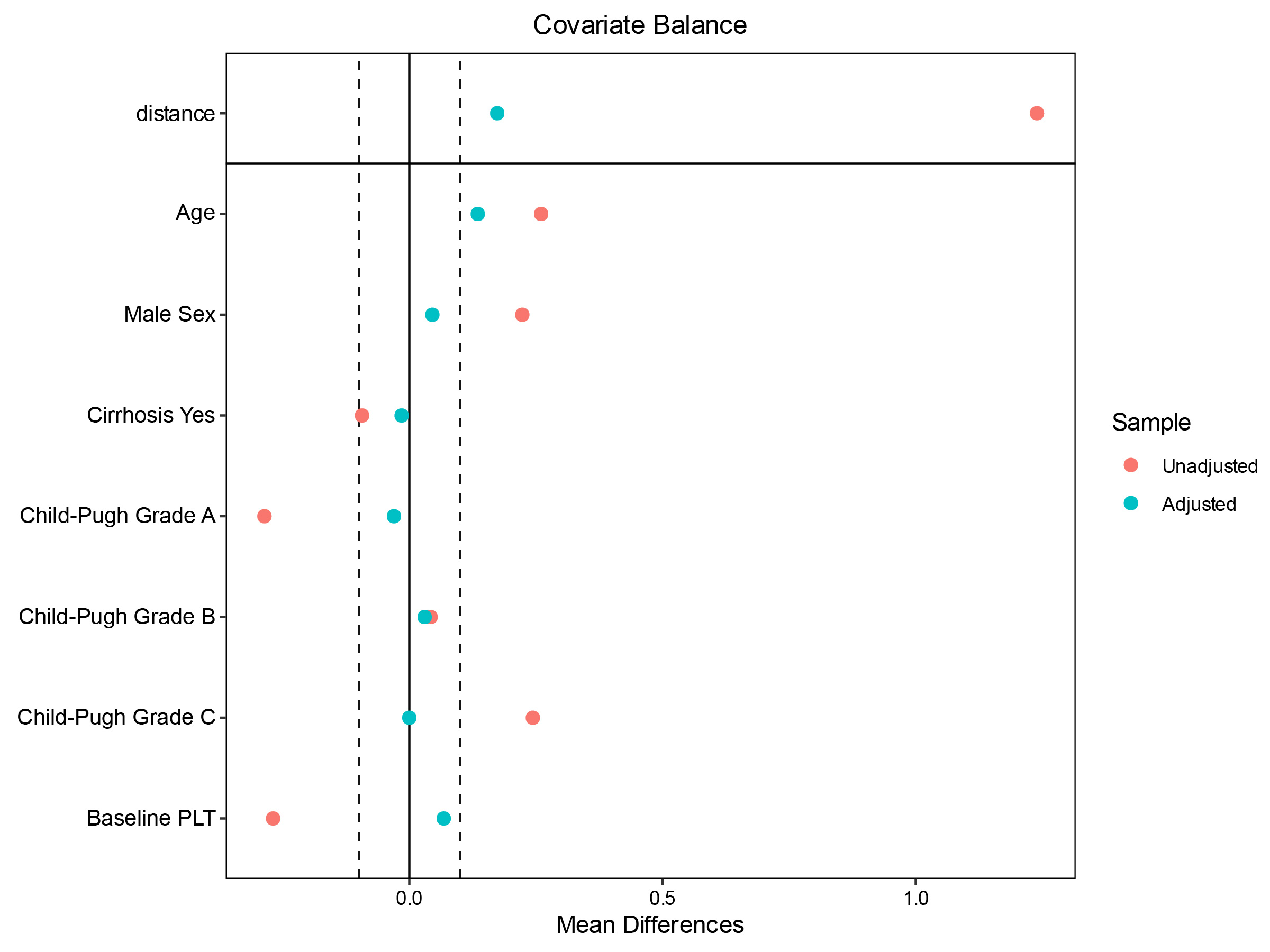

Supplement: Supplementary file 2 [file Image1.jpeg]
