# Supplementary material for: Recombinant human thrombopoietin for chronic liver disease-associated thrombocytopenia with or without concomitant infections: a real-world retrospective study
Source: Front Pharmacol. 2026 Mar 23;17:1732969. doi: 10.3389/fphar.2026.1732969 (PMC13050849; doi:10.3389/fphar.2026.1732969)
Supplement: Supplementary file 3 [file Table2.docx]

**Table S2. Primary efficacy endpoints in the entire cohort and PSM cohort**

| **Subgroup/Parameter** | **Total** | **Concomitant infection** | **Non-concomitant infection** | ***P* value** |
| --- | --- | --- | --- | --- |
| **Entire cohort** | **n=259** | **n=178** | **n=81** |  |
| **Overall** |  |  |  |  |
| Effective rate (%) | 152 (58.7) | 106 (59.6) | 46 (56.8) | 0.676 |
| Time to response (d), median (IQR) | 10 (9-12) | 9 (9-14) | 10 (9-13) | 0.838 |
| **Baseline PLT < 50 × 10^9^/L** | **n=214** | **n=150** | **n=64** |  |
| Effective rate (%) | 132 (61.7) | 93 (62.0) | 39 (60.9) | 0.884 |
| Time to response (d), median (IQR) | 9 (9-11) | 9 (9-12) | 10 (8-13) | 0.825 |
| **50×10^9^/L ≤ Baseline PLT < 75 × 10^9^/L** | **n=45** | **n=28** | **n=17** |  |
| Effective rate (%) | 20 (44.4) | 13 (46.4) | 7 (41.2) | 0.731 |
| Time to response (d), median (IQR) | 14 (10-28)) | 15 (8-Nr) | 10 (10-NR) | 0.766 |
| **PSM cohort** | **n=132** | **n=66** | **n=66** |  |
| **Overall** |  |  |  |  |
| Effective rate (%) | 76 (57.6) | 39 (59.1) | 37 (56.1) | 0.725 |
| Time to response (d), median (IQR) | 10 (9-13) | 10 (8-15) | 11 (10-14) | 0.442 |
| **Baseline PLT < 50 × 10^9^/L** | **n=107** | **n=52** | **n=55** |  |
| Effective rate (%) | 64 (59.8) | 32 (61.5) | 32 (58.2) | 0.723 |
| Time to response, median (IQR), days | 10 (9-12) | 9 (8-14) | 11 (8-15) | 0.445 |
| **50×10^9^/L ≤ Baseline PLT < 75 × 10^9^/L** | **n=25** | **n=14** | **n=11** |  |
| Effective rate (%) | 12 (48) | 7 (50) | 5 (45.5) | 0.821 |
| Time to response (d), median (IQR) | 10 (10-NR) | 15 (8-NR) | 10 (10-NR) | 0.75 |
